# Proteomic analysis of plasma proteins from patients with cardiac rupture after acute myocardial infarction using TMT-based quantitative proteomics approach

**DOI:** 10.1186/s12014-024-09474-9

**Published:** 2024-03-01

**Authors:** Jingyuan Hou, Qiaoting Deng, Xiaohong Qiu, Sudong Liu, Youqian Li, Changjing Huang, Xianfang Wang, Qunji Zhang, Xunwei Deng, Zhixiong Zhong, Wei Zhong

**Affiliations:** 1 Research Experimental Center, Meizhou Clinical Institute of Shantou University Medical College, Meizhou, Guangdong 514031 China; 2 Meizhou clinical Medical School, Guangdong Medical University, Meizhou, Guangdong 514031 China; 3 Center for Cardiovascular Diseases, Meizhou People’s Hospital, Meizhou, Guangdong 514031 China; 4 GuangDong Engineering Technology Research Center for Molecular Diagnostics of Cardiovascular Diseases, Meizhou, Guangdong 514031 China

**Keywords:** Proteomics, Plasma, Cardiac rupture, Acute myocardial infarction, Tandem mass tag, Multiple reaction monitoring

## Abstract

**Background:**

Cardiac rupture (CR) is a rare but catastrophic mechanical complication of acute myocardial infarction (AMI) that seriously threatens human health. However, the reliable biomarkers for clinical diagnosis and the underlying signaling pathways insights of CR has yet to be elucidated.

**Methods:**

In the present study, a quantitative approach with tandem mass tag (TMT) labeling and liquid chromatography–tandem mass spectrometry was used to characterize the differential protein expression profiles of patients with CR. Plasma samples were collected from patients with CR (*n* = 37), patients with AMI (*n* = 47), and healthy controls (*n* = 47). Candidate proteins were selected for validation by multiple reaction monitoring (MRM) and enzyme-linked immunosorbent assay (ELISA).

**Results:**

In total, 1208 proteins were quantified and 958 differentially expressed proteins (DEPs) were identified. The difference in the expression levels of the DEPs was more noticeable between the CR and Con groups than between the AMI and Con groups. Bioinformatics analysis showed most of the DEPs to be involved in numerous crucial biological processes and signaling pathways, such as RNA transport, ribosome, proteasome, and protein processing in the endoplasmic reticulum, as well as necroptosis and leukocyte transendothelial migration, which might play essential roles in the complex pathological processes associated with CR. MRM analysis confirmed the accuracy of the proteomic analysis results. Four proteins i.e., C-reactive protein (CRP), heat shock protein beta-1 (HSPB1), vinculin (VINC) and growth/differentiation factor 15 (GDF15), were further validated via ELISA. By receiver operating characteristic (ROC) analysis, combinations of these four proteins distinguished CR patients from AMI patients with a high area under the curve (AUC) value (0.895, 95% CI, 0.802–0.988, *p* < 0.001).

**Conclusions:**

Our study highlights the value of comprehensive proteomic characterization for identifying plasma proteome changes in patients with CR. This pilot study could serve as a valid foundation and initiation point for elucidation of the mechanisms of CR, which might aid in identifying effective diagnostic biomarkers in the future.

**Supplementary Information:**

The online version contains supplementary material available at 10.1186/s12014-024-09474-9.

## Background

Despite the considerable progress achieved in primary percutaneous coronary intervention (PCI) and medical management, acute myocardial infarction (AMI) remains a leading cause of global morbidity and mortality [1]. Cardiac rupture (CR) is a rare but catastrophic mechanical complication of AMI which encompasses a disease spectrum ranging from rupture of the left ventricular free wall, papillary muscle, or ventricular septum, to, less frequently, atrial rupture [2]. Without treatment, patients with CR may eventually progress to cardiogenic shock or other adverse outcomes, including death. Although the introduction and continuous improvement of early percutaneous revascularization strategies have seen a dramatic decline in the incidence of CR over the past two decades, the mortality rate associated with this severe complication has failed to decrease in parallel [3, 4]. However, these advances are challenged by the progressive aging of the population in China. Moreover, in recent years, the COVID-19 pandemic has delayed primary PCI for patients with AMI [5, 6], and a consequential increase in mechanical complications, including CR, is currently creating concern.

Cardiac rupture mostly presents as an emergency, especially in older people and patients for whom treatment is delayed [7, 8]. Importantly, the severity of the initial symptoms exhibits a substantial degree of heterogeneity. Prompt diagnosis and appropriate management are widely assumed to lead to a better outcome for patients with CR [9, 10]. However, the mortality rate remains high and few patients can be saved. Despite the utmost clinical attention and extensive interest, identifying patients with CR among the large number of patients admitted with chest pain is challenging, especially in the absence of clear clinical signs and available echocardiography [11, 12]. Therefore, it is imperative that more reliable diagnostic and intervention strategies for CR are explored to improve the outcomes of these high-risk patients [13, 14]. Specifically, a better understanding of the molecules and pathways involved in the development of CR may assist in early diagnosis and facilitate the development of new therapeutic strategies.

Proteomics reflects the protein expression changes that occur within cells, tissues, and organisms at different stages and states. In recent years, high-throughput mass spectrometry has been proposed as an efficient quantitative profiling platform for the systematic study of proteomic changes that can assist in deciphering the molecular basis of complex physiological adaptations in various diseases [15–17]. Tandem mass tagging (TMT) is a relatively high-throughput technique with high sensitivity that can accurately quantify disease-related protein changes in serum/plasma profiles and is useful for discovering significant insights into the pathogenesis of various diseases [18–20]. Further, the conventional verification methods used to verify protein expression, such as western blot analysis and enzyme-linked immunosorbent assays (ELISA), are antibody-based approaches and thus, are highly dependent on antibody specificity and have low verification efficiency [21]. In contrast, the multiple reaction monitoring (MRM) strategy exhibits multiplexing capability for the identification of targeted proteins and provides reliable quantitative proteomics results without the presence of appropriate and specific antibodies [22, 23]. Currently, the integration of TMT-based quantitative proteomics with MRM analysis confirmation has become a versatile approach for relative protein quantification and is suitable for exploratory studies of the underlying molecular mechanisms in numerous scientific fields [24–26]. However, the proteomic profile of patients with CR, which is highly desirable, has yet to be elucidated.

In the present study, we first employed a quantitative proteomics approach that relies on TMT-labeling and liquid chromatography–tandem mass spectrometry (LC-MS/MS) to assess the proteomic changes and identify differentially expressed proteins (DEPs) in plasma samples from patients diagnosed with AMI or CR and healthy controls. Our study will provide a foundation for a better understanding of the molecular functions and processes involved in the development of CR.

## Materials and methods

### Study participants and sample collection

The study protocol was performed in line with the recommendations of the Declaration of Helsinki and approved by the Institutional Review Board of Meizhou People’s Hospital (2019-C-113). Before the collection of blood samples, written informed consent was obtained from all participants or their legal guardians. For this study, 37 patients with CR after AMI and 47 patients with AMI alone were enrolled at the Department of Cardiology of Meizhou People’s Hospital between March 2018 and November 2022. All patients were newly diagnosed and screened based on criteria including persistent chest pain, electrocardiogram changes, and high-sensitivity cardiac troponin I testing. The time interval from the onset of chest pain to hospital admission was less than 24 h. The diagnosis of AMI was based on the 2012 Joint ESC/ACCF/AHA/WHF Task Force Third Universal Definition of Myocardial Infarction [27] and confirmed by coronary angiography. CR was confirmed based on clinical symptoms and echocardiogram findings. Patients with a previous history of myocardial infarction, coronary artery bypass surgery, malignant tumor, severe hematological disease, infection, or severe autoimmune disease were excluded. Moreover, patients with congenital heart defects, traumatic cardiac injury or other primary heart diseases were also excluded. Forty-seven healthy individuals with no pre-existing or existing cardiovascular symptoms who underwent physical examinations in the hospital were enrolled as a control group (Con group).

For plasma isolation, blood samples (5 mL) were collected from each participant in BD vacutainer® plus plastic tubes. The plasma was separated by centrifugation at 3,000 × *g* for 20 min at 4 °C within 3 h. The supernatant was then collected and aliquoted into working volumes in sterile cryopreservation tubes and stored at − 80 °C until proteomic analysis.

### Protein extraction and trypsin digestion

Proteomics was first utilized to analyze the plasma samples of the CR, AMI, and Con groups (*n* = 9 per group). The sample preparation procedures and LC-MS/MS methods used were similar to our previously described procedures [28]. Briefly, to reduce the individual differences, three individual plasma samples in each group were pooled together as one biological replicate. Three biological replicates were then generated for each group. To prepare the protein samples, the ProteoMiner Protein Enrichment Kit (Bio-Rad, CA, USA) was used according to the manufacturer’s instructions to deplete highly abundant human proteins. Each tube was loaded with 900 µL of a 0.22 μm-filtered sample and incubated for 2 h at room temperature, after which 100 µL of 1 M sodium citrate and 20 mM of HEPES (pH 7.4) were added. No bead aggregation was observed.

Next, the proteins were desorbed using a two-step elution method. First, the beads were incubated twice with 100 µL of the kit elution reagent [4 M urea, 1% (w/v) CHAPS, 5% (v/v) acetic acid] for 15 min. Then, 100 µL of 6 M guanidine hydrochloride (pH 6.0) was added twice for 15 min. Finally, four elution fractions from each column were pooled and stored at − 80 °C until further analysis. The extracted protein was quantified using a bicinchoninic acid assay (BCA) kit (BioRad, Hercules, USA), and its integrality was confirmed using 10% SDS-PAGE (sodium dodecyl sulfate–polyacrylamide gel electrophoresis). According to the protein concentration, 100 µg of protein from each sample was digested with Trypsin Gold (Promega, Madison, WI, USA). Trypsin was first added at a mass ratio of 1:50 (trypsin:protein) and digested at 37 °C for 16 h, and then added at a mass ratio of 1:100 (trypsin:protein), and the enzymatic hydrolysis was continued for 4 h. After trypsin digestion, the enzymatic reaction was quenched using formic acid (FA, final concentration of 0.5% *v*/*v*). Finally, the resulting peptides were desalted using a Strata X C18 SPE column (Phenomenex, California, USA) and vacuum dried.

### Tandem mass tag labeling

For TMT labeling, digested peptides were reconstituted in 0.5 M tetraethylammonium tetrahydroborate (TEAB) and labeled with isobaric TMT 10-plexed reagents (Thermo Fisher Scientific, Waltham, MA, USA) according to the manufacturer’s protocol. Briefly, one unit of TMT reagent (defined as the amount of reagent required to label 100 µg of protein) was thawed and reconstituted in acetonitrile (ACN), before being mixed with the peptide digest and incubated for 2 h at room temperature. Finally, each interaction mixture was then pooled, desalted, and dried by vacuum centrifugation. The TMT tags 127 N, 127 C and 128 N were used to label peptides from the Con group; tags 128 C, 129 N, and 129 C were used for the AMI group; and tags 130 N, 130 C, and 131 were used for the CR group.

### Liquid chromatography–tandem mass spectrometry (LC-MS/MS)

Equal amounts of labeled peptides were mixed and fractionated using strong cation-exchange chromatography (SCX) on a LC-20AB HPLC Pump system (Shimadzu, Kyoto, Japan). Briefly, the dried peptide mixtures were reconstituted in 2 mL of Buffer A (5% ACN, pH 9.8) and loaded onto an Ultremex SCX column (5 μm, 4.6 mm × 250 mm, Phenomenex, CA, USA). The peptides were eluted at a flow rate of 1 mL/min using a gradient of Buffer B (95% ACN, pH 9.8) starting at 0% for 3 min, 5% for 10 min, then 5–35% for 40 min and 35–95% for 1 min. The system was then maintained at 95% for 3 min, which was decreased to 5% within 1 min, before the column was re-equilibrated at 5% for 10 min. Elution was monitored by measuring the absorbance of the eluent at a wavelength of 214 nm, and the fractions were collected at 1-min intervals. Finally, the SCX peptide fractions were combined into 20 pools, desalted using a Strata X C18 column (Phenomenex), and vacuum dried for LC-MS/MS analysis.

Next, all vacuum-dried fractions were redissolved in Buffer A (2% ACN, 0.1% FA) and centrifuged at 20,000 × *g* for 10 min. The supernatant obtained was loaded onto a HPLC system (Thermo Scientific™ UltiMate™ 3000 UHPLC, Thermo Scientific, Chelmsford, MA, USA) that was connected to a C18 trap column (75 μm × 25 cm, 3 μm, Thermo Fisher Scientific) at a flow rate of 5 µL/min for 8 min. After desalting, the peptides were eluted from the column at a flow rate of 300 nL/min using a gradient of Buffer B (98% ACN, 0.1% FA) rising from 5 to 25% over 40 min, 25–35% over 2 min, 35–80% over 2 min, and then remaining at 80% for 2 min. Finally, it was ramped to 5% Buffer B for another 8 min. The column was re-equilibrated with 5% B for 10 min before subsequent runs.

After liquid phase separation, the peptides were subjected to a nano-electrospray-ionization source followed by tandem mass spectrometry (MS/MS) in a Q Exactive HF X mass spectrometer (Thermo Fisher Scientific) that was coupled online to the ultra-performance liquid chromatography system. The main parameter settings were as follows: The electrospray voltage applied was 2.0 kV. The primary MS scan (m/z) range was set as 350 to 1500 for a full scan, and any intact peptides were detected in an orbitrap at a resolution of 60,000. Peptides were selected for MS/MS analysis with the normalized collision energy set at 30 eV, and the ion fragments were detected in the orbitrap at a resolution of 15,000. A data-dependent procedure that alternated between one MS scan followed by 20 MS/MS scans was applied for the 20 most intense precursors above a threshold ion count of 10,000 in the MS survey scan at a 30.0 s dynamic exclusion. Automatic gain control was set at 3E6. The fixed first mass was set as 100 m/z.

### Protein identification and quantification

The original MS/MS raw data were processed with Proteome Discoverer 1.4 (Thermo Fisher Scientific, San Jose, CA, USA). Tandem mass spectra were searched using the Mascot search engine (version 2.3.02; Matrix Science, Boston, MA, USA) against the UniProt *Homo sapiens* database (date 201,905, with 20,358 protein sequences) concatenated with a reverse decoy database. The Mascot search parameters applied were as follows: Type of search = MS/MS Ion search; Enzyme specificity = Trypsin; Maximum missed cleavages = 1; Mass values = monoisotopic; Fixed modifications = Carbamidomethyl (C), TMT10plex (N-term), TMT10plex (K); Variable modification = Oxidation (M), TMT10plex (Y); Peptide mass tolerance = 20 ppm; Fragment mass tolerance = 0.05 Da. Each confident protein identification involved at least one unique peptide. Quantitative analysis of the peptides was performed using the IQuant software [29]. The false discovery rate was set at 1% for both the peptide spectrum match-level and the protein-level using a Mascot Percolator algorithm. Only proteins containing at least two peptides were considered for further protein quantification. Proteins with a fold change of > 1.20 or < 0.83 with *p* < 0.05 were considered to be significantly differentially expressed.

### Bioinformatics analyses

Functional analysis of DEPs was performed using Gene Ontology (GO) annotations (http://www.geneontology.org/), eukaryotic of orthologous groups (KOG) classification (http://www.ncbi.nlm.nih.gov/KOG/), and the Kyoto Encyclopedia of Genes and Genomes (KEGG) database (http://www.genome.jp/kegg/pathway.html). There are three main GO annotation categories: biological process (BP), cellular component (CC), and molecular function (MF). Fisher’s exact tests were performed for the GO annotation and KEGG pathway enrichment analyses, and only functional categories and pathways with *p* < 0.05 were considered significant.

### Validation of DEPs by MRM

To verify the expression levels of the different proteins obtained through the TMT-labeling proteomic analysis, ten proteins were selected for further quantification by MRM analysis. Samples were digested as described above and spiked with 50 fmol of β-galactosidase for data normalization. Then, MRM analyses were carried out using a QTRAP 6500 mass spectrometer (AB SCIEX, Framingham, MA, USA) equipped with the LC-20AD nanoHPLC system (Shimadzu, Kyoto, Japan). The mobile phase consisted of Buffer A (0.1% aqueous formic acid) and Buffer B (98% ACN with 0.1% FA). The peptides were separated on a C18 column (75 μm × 15 cm, 3.6 μm) at 300 nL/min and eluted using a gradient of Buffer B rising from 5 to 30% over 38 min, 30–80% over 4 min, and then remaining at 80% for 8 min.

For the QTRAP 6500 mass spectrometer, a spray voltage of 2400 V, nebulizer gas of 23 psi., and a dwell time of 10 ms were applied. Multiple MRM transitions were monitored using a unit resolution in both the Q1 and Q3 quadrupoles to maximize the specificity. For the MRM analysis, Skyline software [30] was used on the obtained data to integrate the raw file generated by QTRAP 6500 (SCIEX, Framingham, MA, USA), and an iRT strategy [31] was applied to define the chromatography of a given peptide against a spectral library.

### Validation of DEPs by ELISA

To further validate the identified DEPs, ELISA was performed to measure plasma proteins concentrations according to the manufacturer’s instructions. The concentrations of C-reactive protein (CRP), heat shock protein beta-1 (HSPB1), vinculin (VINC) and growth/differentiation factor 15 (GDF15) were respectively quantified using a Human CRP Quantikine ELISA Kit (DCRP00, R&D Systems), HSPB1 Human ELISA Kit (EHHSPB1, Thermo Fisher Scientific), Human Vinculin ELISA Kit (LS-F34925, LifeSpan Biosciences) and Human GDF15 ELISA Kit (KIT10936, Sino Biological).

### Statistical analysis

Statistical analyses were performed using IBM Statistical Program for Social Sciences (SPSS) software version 22.0 (IBM Corp. New York, USA). Continuous variables was analysed using one-way analysis of variance (ANOVA) followed by the least significant difference (LSD) *post hoc* test. Categorical variables were compared using the chi-squared test or Fisher’s exact test. Receiver operating characteristic (ROC) curve analysis was generated, and the area under the curve (AUC) was calculated. A value of *p* < 0.05 was considered statistically significant.

## Results

### Characteristics of the study population

**Fig. 1:**
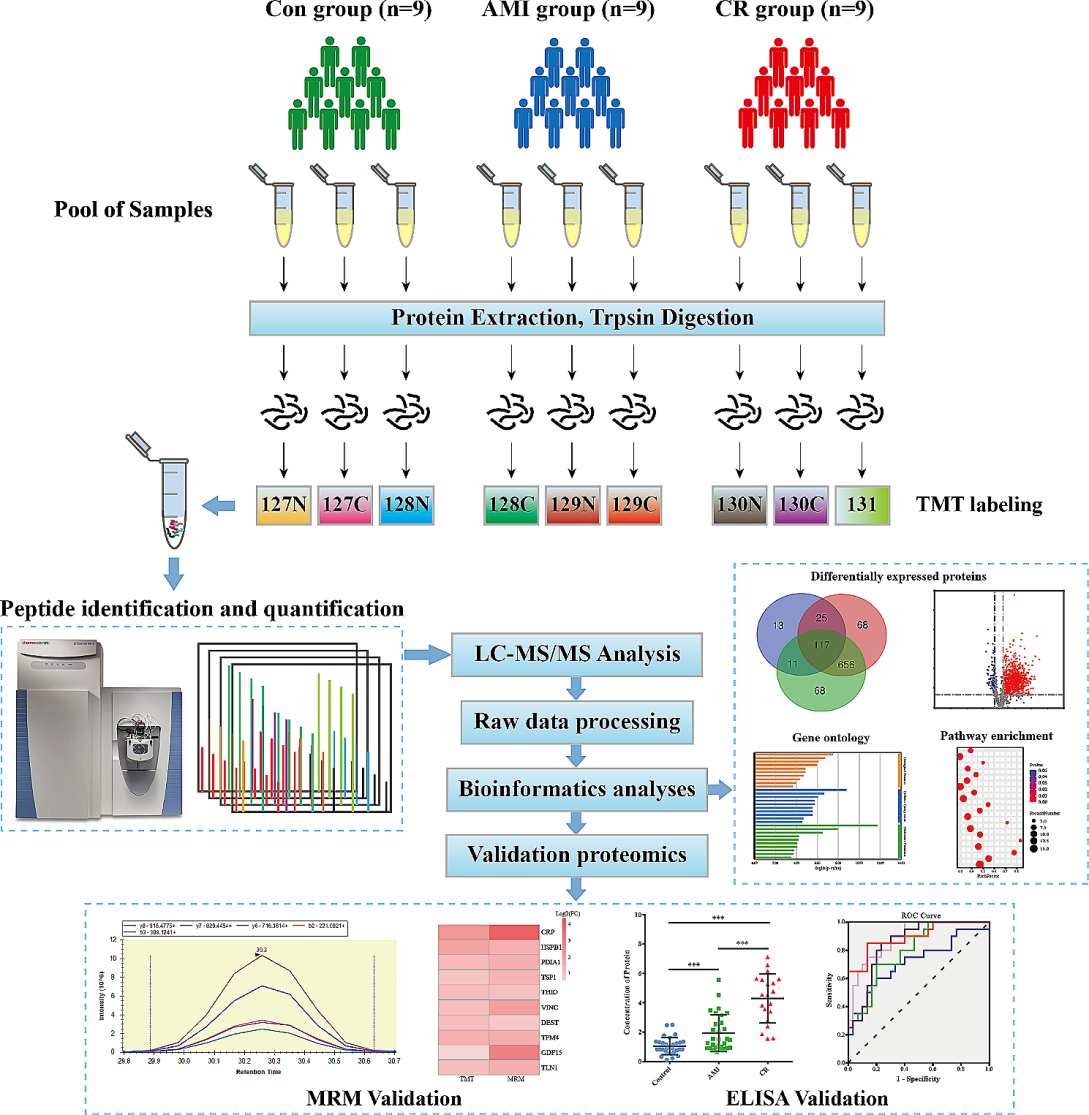
Schematic workflow of tandem mass tag-based quantitative proteomic analysis. The tandem mass tag-based quantitative proteomic analysis was performed on pooled plasma samples. Three independent plasma samples in each group were pooled together as one biological replicate; three biological replicates were generated for each group. After proteomics and bioinformatics analyses, we selected several proteins for the verification stage using multiple reaction monitoring (MRM) analysis and ELISA

To investigate the global proteomic profiles of plasma in the CR, AMI, and Con groups, we performed a quantitative proteomic analysis on plasma samples. The overall experimental strategy and simplified workflow are presented in Fig. 1. During the TMT labeling-based proteomics analysis, three pooled plasma samples were created from 9 CR patients, 9 AMI patients, and 9 healthy controls, respectively, and subsequently profiled in parallel. Then, MRM validation experiments were performed using individual samples from independent cohorts consisting of 8 CR patients, 8 AMI patients, and 8 healthy controls. Finally, plasma samples from additional independent cohorts consisted of 20 CR patients, 30 AMI patients, and 30 healthy controls were used for ELISA validation. The detailed demographic, clinical, and laboratory characteristics of the study participants are presented in Additional file 2: Table S1.

### Identification and quantification of DEPs

**Fig. 2:**
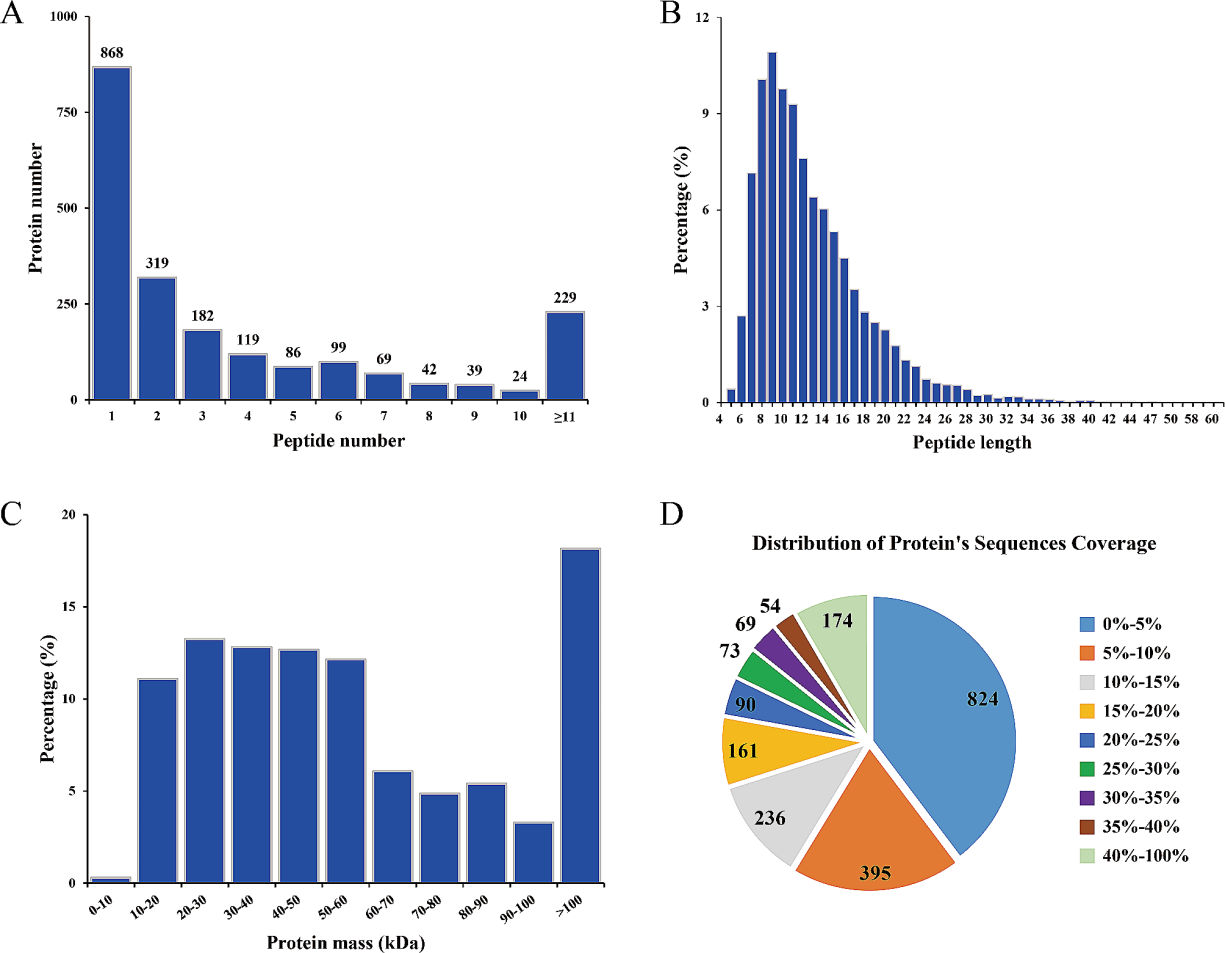
Basic information statistics of the proteome profiled by tandem mass tag proteomics. (**A**) The number of unique peptides of the identified proteins. (**B**) The length distribution of the identified peptides (**C**) The molecular weight distribution of the identified proteins. (**D**) Pie charts classifying the identified proteins according to their sequence coverage

A total of 742,862 spectra were generated from the TMT proteomic analysis (Table 1). Based on the Mascot 2.3.02 search engine analysis, a total of 45,407 spectra were obtained, of which there were 41,051 spectra matched to unique spectra. Ultimately, 10,937 peptides, including 10,420 unique peptides and 2,076 proteins were successfully identified. All identified peptides and proteins are listed in Additional file 3: Table S2 and Additional file 4: Table S3, respectively. The peptide number distribution is shown in Fig. 2A. There were 868, 706, 273, and 229 proteins with 1 peptide, 2–5 peptides, 6–10 peptides, and > 11 peptides, respectively. The length of most peptide fragments was in the range of 7–16 amino acids (Fig. 2B). The protein mass distribution is shown in Fig. 2C. There were 1034 (50%), 659 (32%), and 377 (18%) proteins in the range of 10–50, 50–100, and > 100 kDa, respectively. The distribution of the sequence coverage of the identified proteins is shown in Fig. 2D; as can be seen, approximately 42% of the identified proteins had a sequence coverage of ≥ 10%. Further analysis of the 2,076 identified proteins, 1,208 proteins containing at least 2 unique peptides were quantified. A complete list of quantified proteins is available in Additional file 5: Table S4. These results suggested that the quality of our proteomic analysis conformed to the requirements.

**Table 1:** Spectrums, peptides and proteins identified from TMT-based LC-MS/MS

Total spectras	Spectras	Unique Spetras	Peptides	Unique Peptides	Identified proteins	Quantifiable proteins
742,862	45,407	41,051	10,937	10,420	2076	1208

### Analysis of DEPs

**Fig. 3:**
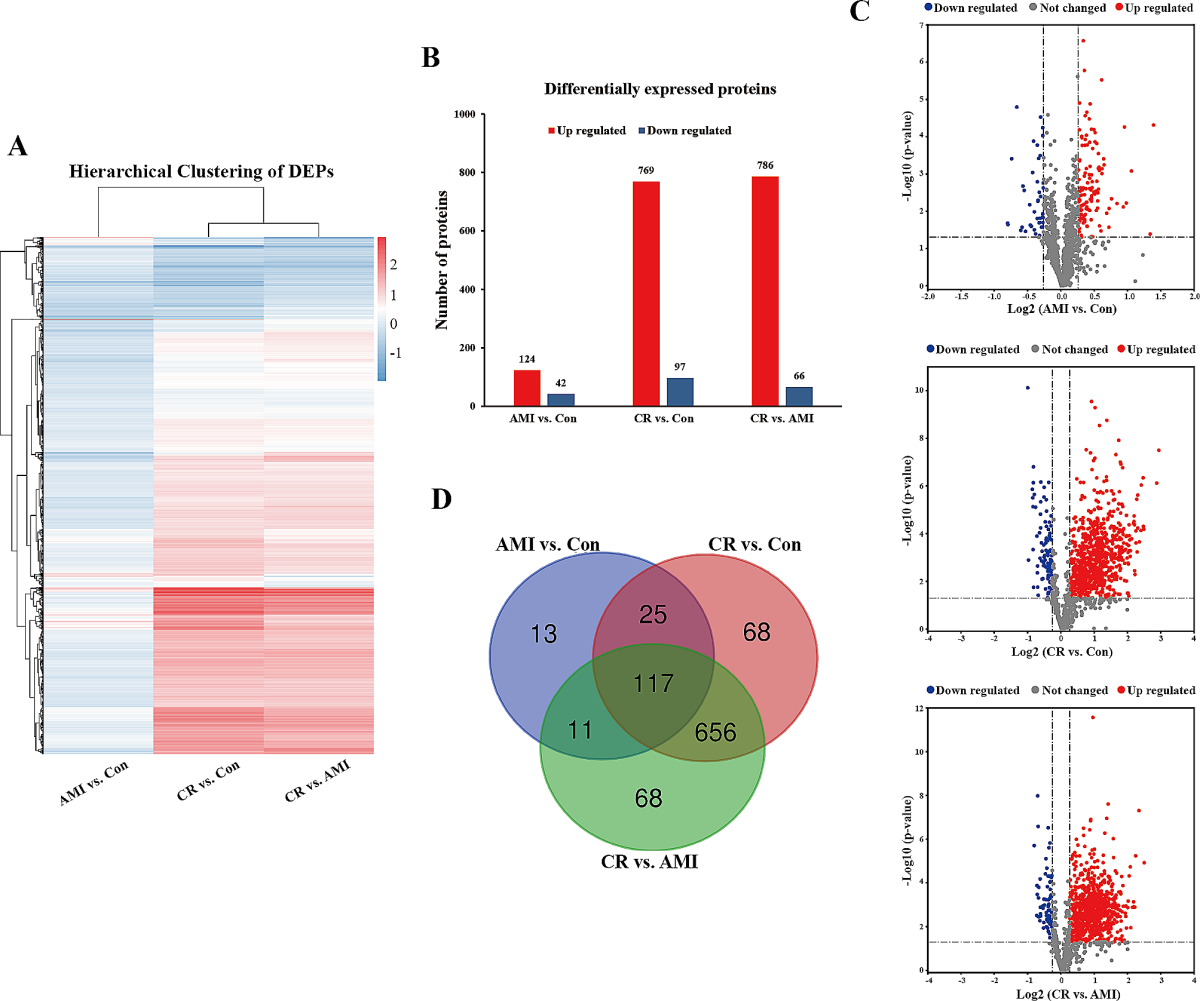
Summary of differentially expressed proteins. (**A**) Hierarchical clustering heat map of the differentially expressed proteins (DEPs). Up-regulated and down-regulated proteins are colored according to the heat map scale (up-regulated: red, down-regulated: blue). (**B**) Histogram of the number distribution of DEPs in different comparison groups. Up-regulated proteins are indicated by red and down-regulated proteins are indicated by blue. (**C**) Volcano plot of DEPs in each comparison group. The x-axis represents Log2 fold change, and the y-axis represents -log10 p value. The red color represents up-regulated proteins; the blue color represents down-regulated proteins; the gray color represents not-regulated proteins. (**D**) Venn diagram of the DEPs identified commonly or exclusively in the three comparison groups

In the current study, significantly DEPs were screened based on a fold change of > 1.2 or < 0.83 in relative abundance (*p* < 0.05). According to the above criteria, a total of 958 DEPs were identified between the three groups, and the hierarchical clustering analysis is shown in Fig. 3A. Additionally, a histogram was used to classify the up- and down-regulated proteins, and volcano plots were created to better visualize the significant DEPs between the groups (Fig. 3B and C). Compared with the Con group, 124 proteins were significantly up-regulated and 42 were significantly down-regulated in the AMI group, and 769 proteins were significantly up-regulated and 97 were significantly down-regulated in the CR group. Also, in the CR group relative to the AMI group, there were 786 proteins that were significantly up-regulated and 66 proteins that were significantly down-regulated. Detailed information on the DEPs is listed in Additional file 6: Table S5.

Moreover, the number of common DEPs between across the three groups and the specific DEPs in each group relative to the other are displayed as Venn diagrams in Fig. 3D. From these data, it was clear that samples in both the CR and AMI groups could be clustered into their respective disease type. The number of dysregulated proteins was significantly higher in the CR group than in the AMI group, indicating a marked change in the number of proteins with the severity of the disease. The considerable differences in protein levels between the plasma samples suggest that biological differences exist between patients and may correlate with clinical status or progression, which could potentially pave the way for molecular biological evidence of CR.

### Functional categories of the DEPs

To obtain a better understanding of the categories and specific functions of the DEPs, we further employed database comparison analysis to conduct KOG functional classification of the DEPs. From the function classification results, comparison of the AMI group versus Con group showed that 45 proteins were involved in signal transduction mechanisms, 41 proteins were involved in the cytoskeleton, 22 proteins were involved in general function prediction only, and 22 proteins were involved in post-translational modification, protein turnover, and chaperones (Additional file 1: Fig. S1A). Comparison of the CR group versus Con group revealed that there were 197 proteins involved in signal transduction mechanisms, 107 proteins involved in the cytoskeleton, 119 proteins involved in general function prediction, and 166 proteins involved in post-translational modification, protein turnover, and chaperones (Additional file 1: Fig. S1B). Similarly, comparison of the CR group versus AMI group showed that 191 proteins were involved in signal transduction mechanisms, 113 proteins were involved in general function prediction, 103 proteins were involved in the cytoskeleton, and 170 proteins were involved in post-translational modification, protein turnover, and chaperones (Additional file 1: Fig. S1C).

**Fig. 4:**
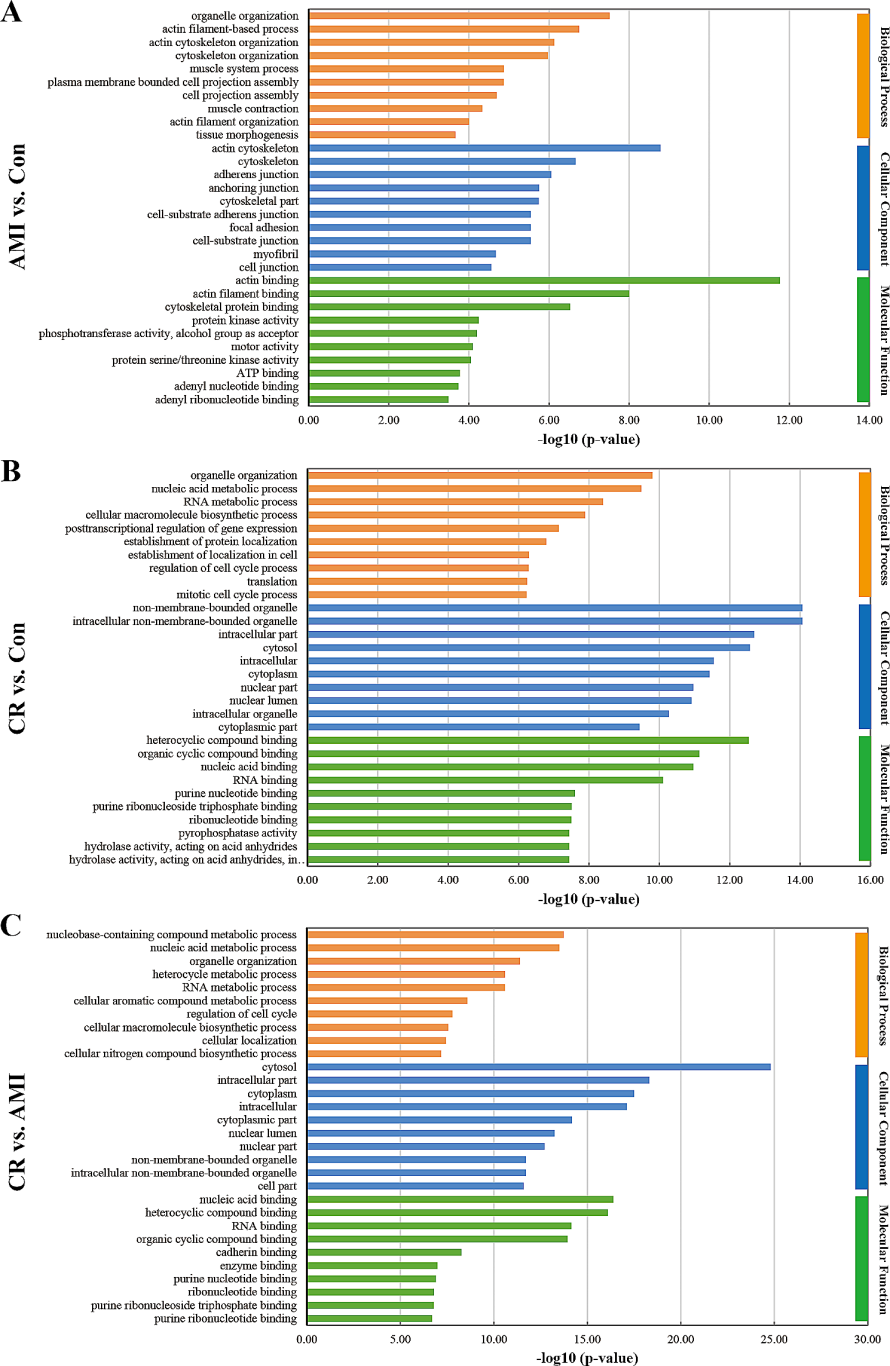
Top 10 statistically significant Gene Ontology (GO) terms (biological processes, cellular components, and molecular functions) compared between the groups (AMI vs. Con, CR vs. Con, and CR vs. AMI). The x-axis indicates the enrichment score [− log10(p value)], and the y-axis indicates the categories of GO terms

To obtain further insights and interpret the biological significance of the DEPs, they were subjected GO annotation and categorized by BP, CC, and MF. The GO terms were ordered according to their *p* values, and the top 10 BP, CC, and MF terms are shown in Fig. 4 and Additional file 7: Table S6. Regarding the AMI group versus the Con group, a total of 277 BP terms, 75 CC terms, and 70 MF terms were obtained through GO functional enrichment analysis (Fig. 4A). The most significantly enriched BP terms were organelle organization, actin filament-based process, actin cytoskeleton organization, cytoskeleton organization, and muscle system process. As for CCs, the proteins were mainly annotated in the actin cytoskeleton, cytoskeleton, adherens junction, anchoring junction, and cytoskeletal part. In the MF category, significantly enriched proteins were involved in actin binding, actin filament binding, cytoskeletal protein binding, protein kinase activity and phosphotransferase activity, and alcohol group as acceptor.

Comparison of the CR group with the Con group revealed 441 BP terms, 105 CC terms, and 76 MF terms. The main enriched BP terms included organelle organization, nucleic acid metabolic process, RNA metabolic process, cellular macromolecule biosynthetic process, and post-transcriptional regulation of gene expression. In terms of CCs, the proteins were predominantly enriched in the non-membrane-bounded organelle, intracellular non-membrane-bounded organelle, intracellular part, cytosol, and intracellular. Regarding MF terms, the proteins were mainly enriched in heterocyclic compound binding, organic cyclic compound binding, nucleic acid binding, RNA binding, and purine nucleotide binding (Fig. 4B).

Regarding the CR group versus the AMI group, the DEPs were mainly enriched in 546 BP terms, 126 CC terms, and 80 MF terms. Regarding BP terms, the proteins were mainly associated with the nucleases-containing compound metabolic process, the nucleic acid metabolic process, organelle organization, the heterocycle metabolic process, and the RNA metabolic process. In the CC category, the proteins were notably enriched in the cytosol, intracellular part, cytoplasm, intracellular, and cytoplasmic part. As for MF terms, the proteins were primarily involved in nucleic acid binding, heterocyclic compound binding, RNA binding, organic cyclic compound binding, and cadherin binding (Fig. 4C).

### KEGG pathway enrichment analysis

**Fig. 5:**
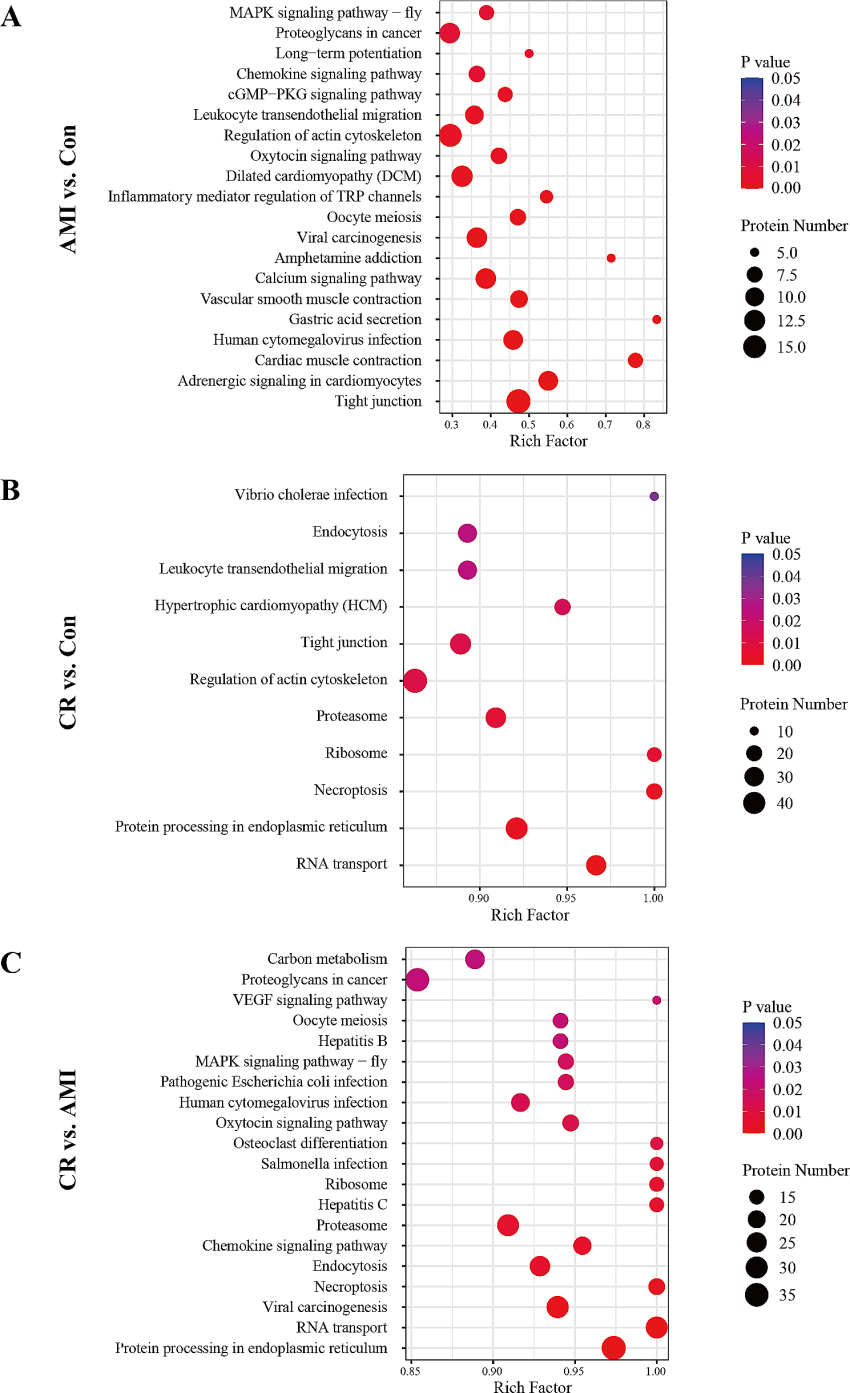
Kyoto Encyclopedia of Genes and Genomes (KEGG) pathway analysis of differentially expressed proteins compared between the groups (**A**) AMI vs. Con, (**B**) CR vs. Con, (**C**) CR vs. AMI. The y-axis shows the significantly enriched KEGG pathways and the x-axis represents the rich factors corresponding to the pathways. The size and color of the nodes represent the number of proteins involved in each KEGG pathway and the p-value of the pathways

Next, to clarify the interaction between DEPs involved in certain biological processes and signaling pathways, pathway enrichment analysis was carried out using the KEGG database. Enriched pathways meeting the standard of *p* < 0.05 were defined as significantly enriched. The related signaling pathways are presented in Additional file 8: Table S7. In the AMI group versus the Con group, 44 statistically significant KEGG pathways were identified, and the DEPs were closely related to tight junction, cardiac muscle contraction, vascular smooth muscle contraction, inflammatory mediator regulation of TRP channels, regulation of actin cytoskeleton, and leukocyte transendothelial migration. These proteins were also mainly involved in adrenergic signaling in cardiomyocytes, the calcium signaling pathway, the cGMP-PKG signaling pathway, and the chemokine signaling pathway. The top 20 KEGG pathways are presented in Fig. 5A.

In the CR group versus the Con group comparison, 11 KEGG enrichment pathways were significantly changed, including RNA transport, protein processing in the endoplasmic reticulum, ribosome, proteasome, necroptosis, regulation of actin cytoskeleton, tight junction, hypertrophic cardiomyopathy (HCM), and leukocyte transendothelial migration (Fig. 5B). In the CR group versus the AMI group comparison, we found that 31 signaling pathways were significantly changed, and the top 20 enriched pathways are shown in Fig. 5C. Several biological pathways were significantly enriched, including protein processing in the endoplasmic reticulum, RNA transport, necroptosis, endocytosis, proteasome, and ribosome, as well as the chemokine signaling pathway. Altogether, these results indicated that biologic events, including dysregulation of specific proteins for each disease type, could be identified. These findings may indicate that different pathogenetic routes are involved in CR and could potentially be used to further understand the complexity of disease progression and to develop diagnostic or therapeutic strategy tools.

### Validation of the DEPs

**Fig. 6:**
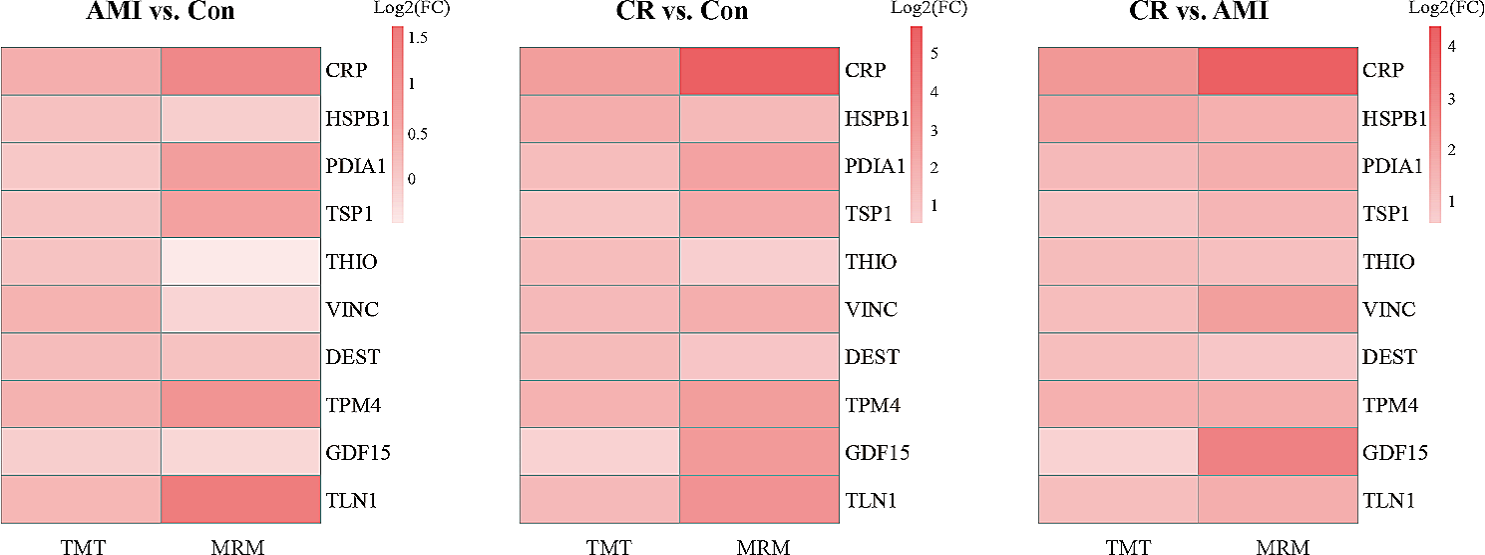
Validation of the target proteins using multiple reaction monitoring (MRM) experiment. Heat map showing the change in abundance of differentially expressed proteins between the groups (AMI vs. Con, CR vs. Con, and CR vs. AMI) using Tandem mass tagging analysis and MRM validation

To validate TMT analysis results, we used MRM to investigate their abundance in an independent validation cohort (*n* = 8 per group) of individual plasma samples. After considering the fold changes in expression, as well as their distinctive biological significance and potential for clinical relevance, we ultimately selected ten proteins of interest for validation: CRP, HSPB1, protein disulfide-isomerase (PDIA1), thrombospondin-1 (TSP1), thioredoxin (THIO), VINC, destrin (DEST), tropomyosin alpha-4 (TPM4), GDF15, and talin-1 (TLN1). The transition information of the target proteins is shown in Additional file 9: Table S8. The relative expression levels of these 10 proteins followed similar trends to those we had observed through the proteomic approach (Fig. 6, Additional file 10: Table S9), which further confirmed the plausibility and reliability of the TMT data.

**Fig. 7:**
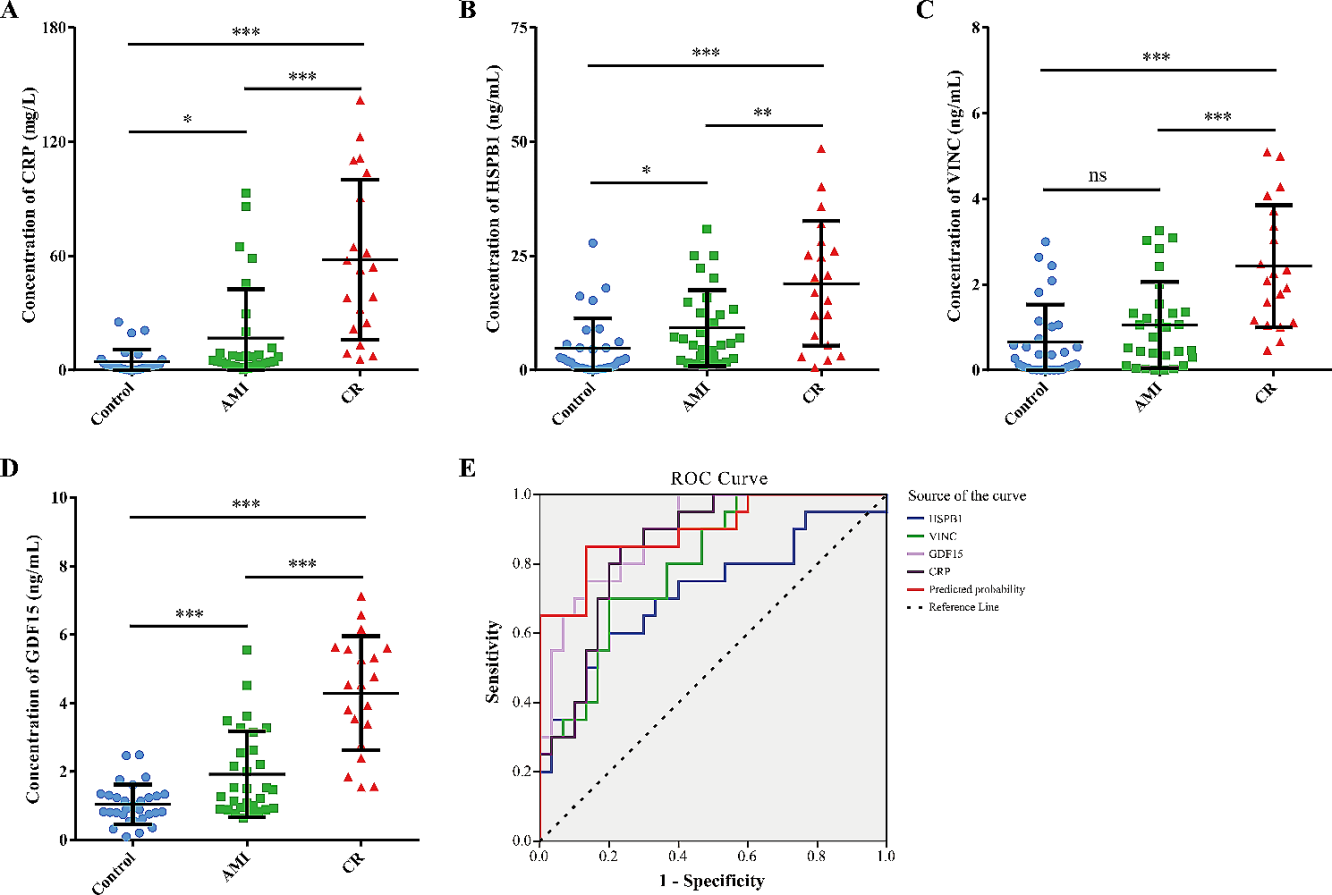
Validation of differentially expressed proteins by ELISA analysis. Proteins concentration in plasma was measured in 20 CR patients, 30 AMI patients, and 30 healthy controls (Con). (**A**) C-reactive protein (CRP). (**B**) Heat shock protein beta-1 (HSPB1). (**C**) Vinculin (VINC). (**D**) Growth/differentiation factor 15 (GDF15). Data are presented as means ± SD. *p* values were showed on the top of each compared groups. *p < 0.01, **p < 0.01, ***p < 0.001, and ns represented not significant. (**E**) ROC curve analysis for discrimination between CR patients and AMI patients

Based on the results of the MRM validation experiment, ELISA assay was performed in additional independent individual plasma samples (CR, *n* = 20; AMI, *n* = 30; Con, *n* = 30). Four candidate proteins i.e. CRP, HSPB1, VINC and GDF15 were selected for verification, as these were obviously increased in the CR patients and we could obtain their commercial ELISA kits. As shown in Fig. 7, the plasma levels of CRP, HSPB1, VINC and GDF15 exhibited differential expression in the three comparisons. Among them, the plasma levels of these protein were significantly higher in CR compared to controls and AMI patients, and variation trends are consist in three groups. Additionally, the ELISA data for these four proteins were then used to confirm the diagnostic efficiency to distinguish CR patients from AMI patients using ROC analysis. AUC values of CRP, HSPB1, VINC, and GDF15 were 0.852 (95% CI, 0.748–0.956, *p* < 0.001), 0.718 (95% CI, 0.566–0.871, *p* = 0.009), 0.797 (95% CI, 0.676–0.918, *p* < 0.001), and 0.887 (95% CI, 0.798–0.976, *p* < 0.001), respectively. Furthermore, the four protein together presented a higher AUC value of (0.895, 95% CI, 0.798–0.976, *p* < 0.001) than that of a single protein. Our results implied that these proteins may serve as biomarkers for early screening and diagnosis of CR.

## Discussion

Cardiac rupture is a globally rare but devastating complication of AMI [2]. Due to its complex pathophysiological mechanism, CR still poses a clinical challenge to treat. Therefore, timely recognition and management are important [9, 12]. Proteomic approaches have been widely used to identify DEPs as potential targets for disease prevention, diagnosis, and therapy [32, 33]. Proteomics might represent a promising approach to uncovering the complex pathological processes associated with CR at the global protein level, a better understanding of which may improve the management of patients with CR as well as disease prevention. To the best of our knowledge, the present study is the first to use a TMT-based quantitative proteomic approach to reveal the plasma proteomic changes in patients with AMI and CR, and healthy controls. We discovered and confirmed DEPs in patients with CR, which subsequent bioinformatics analysis revealed to participate in numerous biological processes and pathways. Thus, this pilot study is expected to present a valid foundation and initiation point for further investigation of the common molecular mechanisms of CR, and thereby provide evidence on diagnostic biomarkers or potential therapeutic targets.

This study was designed to describe the proteomic profile of plasma in patients with CR. There is general agreement that the CR is an important cause of death in the acute phase after AMI. Considering that there may be some similarities in clinical symptoms and pathological mechanisms between CR and myocardial infarction [34], the question is raised as to whether similar changes occur at the protein level. Since CR develops from AMI in most cases, it would be noteworthy to establish whether critical changes in plasma protein composition can distinguish CR from AMI and reflect the development and progression of CR. Therefore, in this study, we carried out a comparative proteomic analysis to identify pivotal functional proteins and pathways in patients with AMI and those with CR. This integrative proteomic analysis protocol is thereby considered to be beneficial in providing a comprehensive view of the protein profiles of patients with CR and informative conclusions for further understanding the possible pathological mechanism of this disease.

First, it is necessary to understand the differences in protein expression between the patients with AMI and the healthy controls. After comparing the AMI and Con groups, our proteomic analysis identified 166 DEPs. As expected, functional analysis showed that these proteins were related to AMI pathogenic processes such as cardiac muscle contraction, vascular smooth muscle contraction, regulation of actin cytoskeleton, leukocyte transendothelial migration, and tissue morphogenesis. Pathway analysis suggested that various pathways, including adrenergic signaling in cardiomyocytes, the calcium signaling pathway, the cGMP-PKG signaling pathway, inflammatory mediator regulation of TRP channels, and the chemokine signaling pathway, may play important roles in the progression of AMI. For example, the first step in AMI development is myocardial ischemia as a result of reduced coronary flow and a deficient oxygen supply. A natural compensatory mechanism may be that when myocardial ischemia occurs, cardiomyocyte contraction is augmented by β-adrenergic signaling. Adrenergic regulation of the calcium signaling pathway can enhance Ca^2+^ entry and increase contractility in both atrial and ventricular cardiomyocytes, and is thereby responsible for both sustainable cardiac function and blood pressure [35, 36]. Previous studies have revealed that disruption of intracellular calcium homeostasis leads to endoplasmic reticulum stress, aggravating ischemic injury of cardiomyocyte contractility and worsening cardiac function outcomes [37, 38]. Similarly, improving calcium homeostasis in vascular smooth muscle is also linked to the promotion of Ca^2+^ influx and enhanced contractility of arterial myocytes. Previous reports have shown that the cGMP-PKG signaling pathway plays an important role in the modulation of intracellular Ca^2+^ concentration, which has been linked to cardioprotection [39]. Additionally, numerous reports have shown that an excessive inflammatory response can stimulate leukocytes to chemotaxis to the vascular wall and infiltrate the endothelium, generating a milieu that supports the development of atherosclerosis and vascular pathologies [40, 41]. Furthermore, ischemia and reperfusion in the injured heart has been shown to cause irreversible tissue damage; the area is subsequently repaired by fibrotic scar tissue, leading to ventricular remodeling [42]. Combined with the above results, this suggests that the altered proteins identified in our analysis are involved in various biological processes and pathways that have previously been linked to the pathophysiology of atherosclerosis or myocardial injury, which provides a direction for the investigation of novel targets in clinical practice. Additionally, it is reassuring that our findings are in line with those reported in recent literature [43]. Specifically, their study revealed a notable alteration in 33 proteins among AMI patients when compared to the control group, and three of these proteins are encompassed within our list of DEPs in AMI plasma. These findings also confirmed that our patient and control groups were appropriately utilized and that the TMT proteomics approach is effective for accurately reflecting the perturbation of protein expression following AMI.

Despite the considerable efforts that have been made in recent years to investigate the complicated molecular mechanisms of CR, identification of the potential underlying mechanisms is still in its infancy. Blood is in direct contact with all tissues, and the pathological changes of CR are likely to be reflected in the plasma proteomic profiles of patients. In this study, analysis of the proteomic changes in patients with CR identified 866 and 852 DEPs in comparison with the healthy controls and patients with AMI, respectively. Our results demonstrated that the alterations in the plasma protein profiles of patients with CR were strikingly dramatic. The present proteomic analysis offers convincing evidence that patients with CR have a distinct proteomic profiles from healthy controls and patients with AMI. Of the DEPs, most were up-regulated. We performed functional enrichment analyses to describe the potential roles of the DEPs in the pathogenesis of CR. The altered proteins were found to be involved in a variety of biological processes, such as organelle organization, biosynthetic and metabolic process, and the cell cycle process. We identified several biological pathways related to potential mechanisms; these mainly included RNA transport, protein processing in the endoplasmic reticulum, ribosome, proteasome, regulation of the actin cytoskeleton, tight junction, endocytosis, leukocyte transendothelial migration, and necroptosis. These findings provide several novel clues about the aberrant biological processes and signaling pathways that may play a critical role in the pathogenesis of CR.

The occurrence of CR is an unanticipated event that can occur suddenly, followed by the rapid development of hemodynamic deterioration and cardiac arrest. Some patients with CR may succumb almost instantaneously with rapid, irreversible [7]. Blood plasma contains numerous proteins that are involved in the cytoskeletal structure or metabolic processes and are required for normal cellular maintenance. Under normal physiological conditions, differences in the abundance levels of these proteins are tightly regulated [44]. Thus, it might be speculated that patients with CR inevitably undergo a number of biological system changes, including metabolic, cardiovascular, hematologic, and respiratory changes, in order to adapt and maintain the fundamental conditions and substrates required for activities and functions in the acute phase [44–46]. Interestingly, the present results support this scenario. They showed that the identified proteins participate in numerous biological processes, such as the metabolic process, the cellular macromolecule biosynthetic process, posttranscriptional regulation of gene expression, and the cell cycle process, as well as regulation of actin cytoskeleton and tight junction. We believe this participation is pertinent to the progression of CR. Additionally, maintenance of cardiac structure and function requires precise control of protein synthesis, processing, and degradation. The endoplasmic reticulum (ER) is a cellular organelle that is largely responsible for responsible for protein folding, calcium homeostasis, and lipid biosynthesis [47]. When the blood flow cannot satisfy the metabolic demand of the myocardium and heart muscle damage occurs, cells undergo enormous morphological and biochemical changes involving the cell cycle, metabolism, and intracellular protein denaturation, and misfolding rates increase, which eventually leads to ER stress [48–50]. In fact, ER stress is understood to be a common pathological process shared by many cardiomyopathy-related disorders. Interestingly, recent evidence shows that AMI induces ER stress and provokes cardiac apoptosis and fibrosis, culminating in CR [51]. Similarly, in the present study, the identified proteins were found to be highly enriched in protein processing in the endoplasmic reticulum. The pathogenesis of CR is undoubtedly multifactorial, and an excessive inflammatory response may be a vital pathophysiological mechanism of rupture [52]. Massive apoptosis and necrosis of cardiomyocytes in response to infarction trigger an inflammatory response that contributes to infarct-induced CR [53, 54]. Therefore, it is not surprising that the leukocyte transendothelial migration and necroptosis signaling pathways were enriched in the KEGG pathway analysis in this study. Indeed, several experimental and clinical studies have reported inflammatory cell infiltration of the infarcted myocardium, especially in the necrotic areas [55, 56]. Also, high blood cell parameters in patients with AMI are associated with a poor survival rate, and some of them are independently related to the risk of CR [57–59].

Finally, we selected ten proteins that are involved in the key processes mentioned above for MRM analysis in individual plasma samples. The results were consistent with the proteomics results. Furthermore, four proteins i.e., CRP, HSPB1, VINC, and GDF15 were successfully validated by ELISA analysis. By ROC curve analysis, the results showed that combining four proteins lead to a higher AUC value, suggesting that these protein possesses the potential power in discriminating CR from AMI. Among these proteins, CRP is well established as one of the most reliable markers of inflammatory response. Previous clinical studies have shown that the peak CRP value is an important predictor of CR after AMI [60–62]. HSPB1, also named Hsp27, has been shown to be involved in cellular adaptation to stress and the maintenance of homeostasis, and its expression can be induced in response to inflammation, oxidative stress, or ischemia [63]. Increased plasma levels of HSPB1 have been observed in patients with acute coronary syndrome [64]. Another study demonstrated that HSPB1 is up-regulated and phosphorylated in the platelets of patients with ST-elevation myocardial infarction [65]. Recently, a study in mice and primary cardiomyocytes demonstrated that cardiomyocyte-specific knockout of HSPB1 is closely associated with increased nuclear factor-κB activation and excessive inflammation, and ultimately results in adverse remodeling and CR [66].

Previously, VINC has been shown to regulate the mechanochemical pathway of the extracellular matrix and cytoskeleton, which contributes to the structural and functional integrity of the heart [67, 68]. Imbalance in the degradation and synthesis of extracellular matrix can progress to CR, highlighting that this protein is an interesting target for further investigation. Also, GDF-15 is a stress-response biomarker associated with several types of cardiovascular disease [69]. Some preclinical animal studies have shown that GDF-15 can protect mice from CR after myocardial infarction by inhibiting the activation of chemokine-triggered leukocyte integrin [70, 71]. In this study, the proteomic clues and validation results, together with DEPs observed increase in abundance in CR consistent with extensive literature, reinforce confidence in the use of our TMT-MRM approach to identify plasma biomarkers. Our results strongly support their importance in the pathogenesis of CR as potential biomarker candidates and therapeutic targets. Nevertheless, validation of these proteins in a larger patient cohort may produce clinically applicable biomarkers of CR disease.

Our study has some limitations that are worth mentioning. Firstly, the sample size for the proteomic analysis in this study was relatively small; therefore, our results should be viewed as preliminary. Another potential limitation of the present study is its cross-sectional design. For more authentic results, future studies, ideally involving a larger population and longitudinal follow-up cohorts, are required. Moreover, it remains unclear whether the DEPs identified contribute directly to the pathogenesis of CR, and further validation of these proteins will enhance the validity and practicality of our results.

## Conclusions

In conclusion, this is the first proteomic research to investigate alterations in the protein levels in patients with CR. The results indicate that a highly dynamic regulation in the plasma of these patients. The DEPs were enriched in several different biological processes and signaling pathways, including RNA transport, protein processing in endoplasmic reticulum, necroptosis, and leukocyte transendothelial migration. Using the MRM and ELISA methodology, four proteins including CRP, HSPB1, VINC, and GDF15 were validated and may serve as a potential diagnostic biomarker for CR disease in AMI patients. Our preliminary results provide a comprehensive plasma proteome inventory of patients with CR, which may offer novel insights and a better understanding of the molecular mechanisms of CR, thereby facilitating the discovery of new diagnostic and therapeutic strategies.

### Electronic supplementary material

Below is the link to the electronic supplementary material.

Supplementary Material 1: Figure **S1**. Distribution of the functional classification of differentially expressed proteins based on eukaryotic of orthologous groups classification. (A) AMI vs. Con; (B) CR vs. Con; (C) CR vs. AMI

Supplementary Material 2: Table **S2**. Demographic and clinical characteristics of the study population

Supplementary Material 3: Table **S3**. Information of all identified peptides

Supplementary Material 4: Table **S4**. Information of all identified proteins

Supplementary Material 5: Table **S5**. Information of quantification proteins

Supplementary Material 6: Table **S6**. Differentially expressed protein information among the three comparison groups

Supplementary Material 7: Table **S7**. GO analysis of the differentially expressed proteins among the three comparison groups

Supplementary Material 8: Table **S8**. Pathway enrichment analysis of the differentially expressed proteins among the three comparison groups

Supplementary Material 9: Table **S9**. The transition information of the validated proteins in MRM

Supplementary Material 10: Table **S10**. Multiple reaction monitoring (MRM) validation results of the ten selected proteins

## Data Availability

The datasets supporting the conclusions of this article are included within the article and its additional files.

## Abbreviations

CR: Cardiac rupture
AMI: Myocardial infarction
TMT: Tandem mass tagging
MRM: Multiple reaction monitoring
ELISA: Enzyme-linked immunosorbent assay
DEPs: Differentially expressed proteins
CRP: C-reactive protein
HSPB1: Heat shock protein beta-1
VINC: Vinculin
GDF15: Growth/differentiation factor 15
ROC: Receiver operating characteristic
AUC: Area under the curve
PCI: Primary percutaneous coronary intervention
LC-MS/MS: Liquid chromatography–tandem mass spectrometry
SCX: Strong cation-exchange chromatography
GO: Gene Ontology
KOG: Eukaryotic of orthologous groups
KEGG: Kyoto Encyclopedia of Genes and Genomes
BP: Biological process
CC: Cellular component
MF: Molecular function
HCM: Hypertrophic cardiomyopathy
PDIA1: Protein disulfide-isomerase
TSP1: Thrombospondin-1
THIO: Thioredoxin
DEST: Destrin
TPM4: Tropomyosin alpha-4
TLN1: Talin-1
ER: Endoplasmic reticulum
