# Supplementary figures and images for: Proteomic analysis of plasma proteins from patients with cardiac rupture after acute myocardial infarction using TMT-based quantitative proteomics approach
Source: Clin Proteomics. 2024 Mar 1;21:18. doi: 10.1186/s12014-024-09474-9 (PMC10908035; doi:10.1186/s12014-024-09474-9)

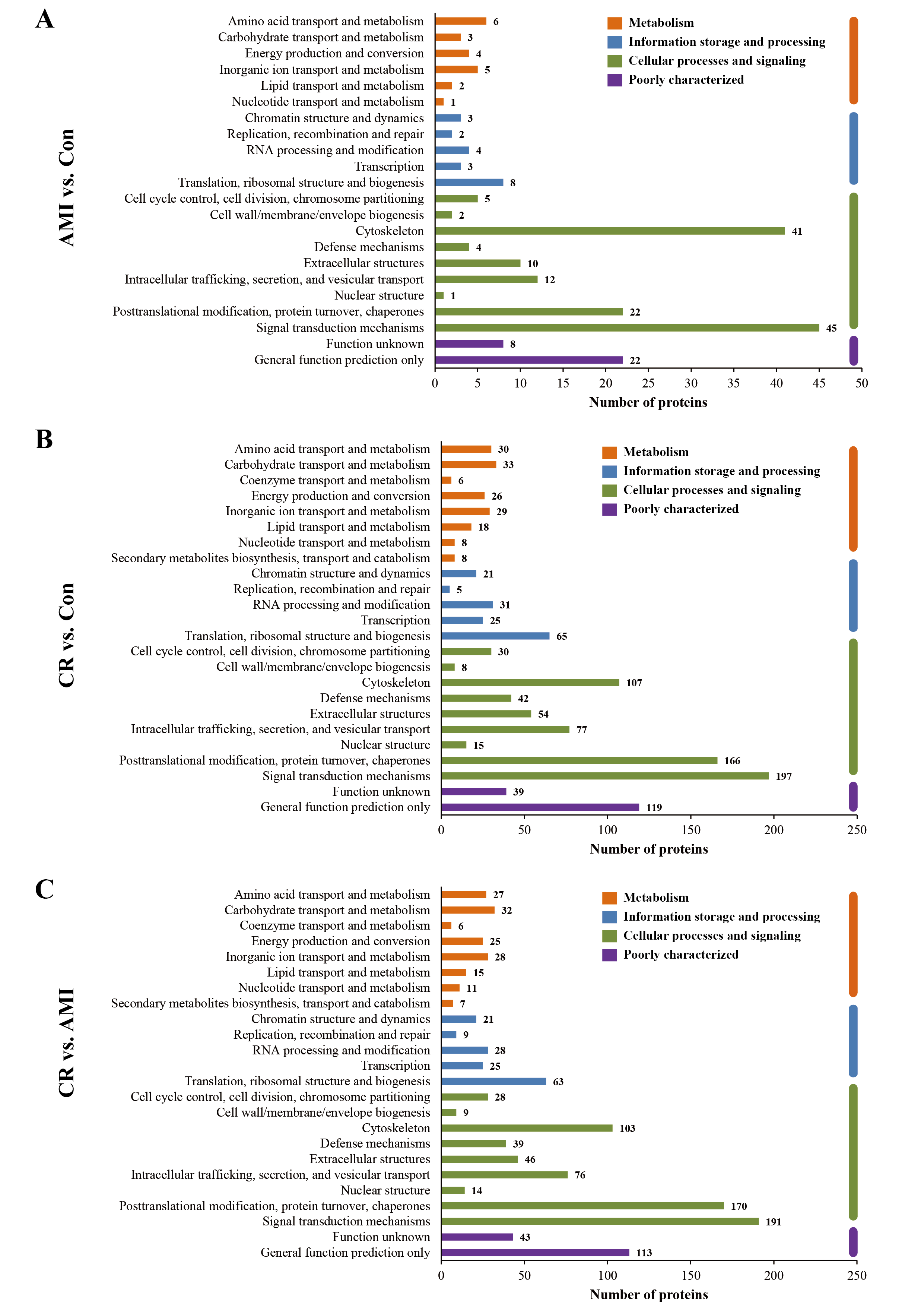

Supplement: Supplementary file 1 — Supplementary Material 1: Figure S1. Distribution of the functional classification of differentially expressed proteins based on eukaryotic of orthologous groups classification. (A) AMI vs. Con; (B) CR vs. Con; (C) CR vs. AMI [file 12014_2024_9474_MOESM1_ESM.tif]
