# Supplementary material for: Proteomic analysis of plasma proteins from patients with cardiac rupture after acute myocardial infarction using TMT-based quantitative proteomics approach
Source: Clin Proteomics. 2024 Mar 1;21:18. doi: 10.1186/s12014-024-09474-9 (PMC10908035; doi:10.1186/s12014-024-09474-9)
Supplement: Supplementary file 2 — Supplementary Material 2: Table S2. Demographic and clinical characteristics of the study population [file 12014_2024_9474_MOESM2_ESM.docx]

**Table S1.** Demographic and clinical characteristics of the study population.

| Variables | Discovery group | | | *P* value | MRM Validation | | | *P* value | ELISA Validation | | | *P* value |
| --- | --- | --- | --- | --- | --- | --- | --- | --- | --- | --- | --- | --- |
|  | CR  (n = 9) | AMI  (n = 9) | Control  (n = 9) |  | CR  (n = 8) | AMI  (n = 8) | Control  (n = 8) |  | CR  (n = 20) | AMI  (n = 30) | Control  (n = 30) |  |
| Age (years) | 77.33±9.53 | 74.44±7.94 | 75.33±8.67 | 0.775 | 73.13±9.43 | 73.50±7.37 | 72.88±7.40 | 0.988 | 70.10±12.11 | 69.37±11.05 | 68.53±6.40 | 0.857 |
| Male, n(%) | 6 (66.7) | 6 (66.7) | 6 (66.7) | 1 | 5 (62.5) | 5 (62.5) | 5 (62.5) | 1 | 13 (65) | 20 (66.7) | 20 (66.7) | 1 |
| Hypertension, n(%) | 5 (55.6) | 5 (55.6) | 1 (11.1) | 0.109 | 5 (62.5) | 5 (62.5) | 3 (37.5) | 0.670 | 10 (50) | 21 (70) | 20 (66.7) | 0.327 |
| Diabetes mellitus, n(%) | 4 (44.4) | 2 (22.2) | 1 (11.1) | 0.418 | 3(37.5) | 0 (0) | 1(12.5) | 0.273 | 5 (25) | 5 (16.7) | 6 (20) | 0.730 |
| Current smoking, n(%) | 4 (44.4) | 3 (33.3) | 3 (33.3) | 1 | 1 (12.5) | 3 (37.5) | 0 (0) | 0.273 | 1 (5) | 13 (43.3) | 5 (16.7) | 0.004 |
| Current drinking, n(%) | 1 (11.1) | 0 (0) | 1 (11.1) | 1 | 0 (0) | 1 (12.5) | 1 (12.5) | 1 | 0 (0) | 4 (13.3) | 0 (0) | 0.038 |
| Dyslipidemia, n(%) | 1 (11.1) | 1 (11.1) | 2 (22.2) | 1 | 2 (25) | 2 (25) | 0 (0) | 0.494 | 2 (10) | 7 (23.3) | 4 (13.3) | 0.476 |
| TG (mmol/L) | 1.40±0.51 | 1.22±0.56 | 1.27±0.69 | 0.791 | 2.12±1.81 | 1.27±0.43 | 1.11±0.22 | 0.157 | 1.40±0.49 | 1.59±0.79 | 1.39±0.65 | 0.482 |
| TC (mmol/L) | 5.55±1.38 | 5.19±0.67 | 4.92±1.17 | 0.497 | 4.97±1.43 | 4.87±1.05 | 4.68±1.04 | 0.883 | 3.89±1.07 | 5.26±1.48 | 4.44±1.06 | 0.001 |
| HDL-C (mmol/L) | 1.44±0.47 | 1.31±0.18 | 1.17±0.31 | 0.278 | 1.27±0.21 | 1.44±0.32 | 1.28±0.36 | 0.450 | 1.02±0.42 | 1.30±0.30 | 1.25±0.31 | 0.021 |
| LDL-C (mmol/L) | 3.25±0.98 | 3.09±0.56 | 3.02±0.86 | 0.823 | 2.34±0.86 | 2.35±0.67 | 2.40±0.48 | 0.982 | 2.15±0.72 | 2.71±1.00 | 2.39±0.74 | 0.083 |
| Apo-A1 (g/L) | 1.25±0.43 | 1.19±0.20 | 1.06±0.30 | 0.436 | 1.15±0.28 | 1.29±0.26 | 1.08±0.33 | 0.361 | 0.88±0.30 | 1.14±0.21 | 1.15±0.23 | 0.001 |
| Apo-B (g/L) | 0.96±0.28 | 0.89±0.20 | 0.91±0.21 | 0.828 | 0.94±0.45 | 0.81±0.21 | 0.80±0.14 | 0.576 | 0.72±0.18 | 0.88±0.28 | 0.74±0.20 | 0.022 |

Data presented are mean ± SD or n (%). Abbreviations: TG, Total triglycerides; TC, Total cholesterol; LDL-C, Low density lipoprotein cholesterol; HDL-C, High density lipoprotein cholesterol; Apo-A1, Apolipoprotein A1; Apo-B, Apolipoprotein B.
