# Supplementary material for: Proteomic analysis of plasma proteins from patients with cardiac rupture after acute myocardial infarction using TMT-based quantitative proteomics approach
Source: Clin Proteomics. 2024 Mar 1;21:18. doi: 10.1186/s12014-024-09474-9 (PMC10908035; doi:10.1186/s12014-024-09474-9)
Supplement: Supplementary file 10 — Supplementary Material 10: Table S10. Multiple reaction monitoring (MRM) validation results of the ten selected proteins [file 12014_2024_9474_MOESM10_ESM.docx]

**Table S9.** Multiple reaction monitoring (MRM) validation results of the ten selected proteins.

| Accession | Protein | Protein Descriptions | AMI/Con | | CR/Con | | CR/AMI | |
| --- | --- | --- | --- | --- | --- | --- | --- | --- |
|  |  |  | TMT | MRM | TMT | MRM | TMT | MRM |
| P02741 | CRP | C-reactive protein | 1.41 | 2.45 | 7.35 | 51.90 | 5.68 | 21.17 |
| P04792 | HSPB1 | Heat shock protein beta-1 | 1.13 | 0.98 | 4.59 | 3.17 | 4.15 | 3.25 |
| P07237 | PDIA1 | Protein disulfide-isomerase | 1.04 | 1.75 | 2.71 | 6.11 | 2.58 | 3.49 |
| P07996 | TSP1 | Thrombospondin-1 | 1.09 | 1.66 | 2.22 | 4.87 | 2.11 | 2.93 |
| P10599 | THIO | Thioredoxin | 1.10 | 0.72 | 2.70 | 1.62 | 2.46 | 2.25 |
| P18206 | VINC | Vinculin | 1.32 | 0.91 | 3.00 | 4.42 | 2.37 | 4.88 |
| P60981 | DEST | Destrin | 1.19 | 1.12 | 2.97 | 2.12 | 2.31 | 1.89 |
| P67936 | TPM4 | Tropomyosin alpha-4 | 1.36 | 2.07 | 3.96 | 7.29 | 3.29 | 3.53 |
| Q99988 | GDF15 | Growth/differentiation factor 15 | 0.98 | 0.89 | 1.46 | 8.31 | 1.50 | 9.32 |
| Q9Y490 | TLN1 | Talin-1 | 1.28 | 3.04 | 3.00 | 10.40 | 2.33 | 3.43 |
